# Blockage of High-Affinity Choline Transporter Increases Visceral Hypersensitivity in Rats with Chronic Stress

**DOI:** 10.1155/2018/9252984

**Published:** 2018-04-04

**Authors:** Chen Zhao, Mengjuan Lin, Yasi Pan, Baoping Yu

**Affiliations:** ^1^Department of Gastroenterology, Renmin Hospital of Wuhan University, Wuhan, China; ^2^Hubei Key Laboratory of Digestive System Diseases, Wuhan, China

## Abstract

**Background:**

Visceral hypersensitivity is a common feature of irritable bowel syndrome. Cholinergic system involves in the development of visceral hypersensitivity, and high-affinity choline transporter (CHT1) is of crucial importance in choline uptake system. However, involvement of CHT1 in visceral hypersensitivity remains unknown. The research aimed to study the CHT1 expression in dorsal root ganglions (DRGs) and the role of CHT1 in visceral hypersensitivity.

**Methods:**

Repetitive water avoidance stress (WAS) was used to induce visceral hypersensitivity in rats. Colorectal distension (CRD) was determined, and the abdominal withdrawal reflex (AWR) and threshold intensity data were recorded to measure the visceral sensitivity. After intraperitoneal injection of hemicholinium-3 (HC-3), the specific inhibitor of CHT1, CRD data were also recorded. The CHT1 expression of DRGs was investigated by Western blotting, immunohistochemistry, and quantitative RT-PCR. Acetylcholine levels in the DRGs were detected by the assay kit.

**Results:**

Repetitive WAS increased the AWR score of CRD at high distension pressure and decreased the mean threshold of rats. The CHT1 expression and acetylcholine concentration of DRG were significantly increased in WAS rats. After the administration of HC-3, the AWR score in WAS group was significantly increased at higher distension pressure while the threshold intensity was significantly reduced compared to the normal saline group. Acetylcholine concentration was significantly lower than the normal saline rats.

**Conclusion:**

Our research firstly reports that CHT1 is overexpressed in noninflammatory visceral hypersensitivity, and blockage of CHT1 can enhance the visceral hypersensitivity. CHT1 may play an inhibitory role in visceral hypersensitivity.

## 1. Introduction

Irritable bowel syndrome (IBS) is a common gut disorder with altered bowel habits, abdominal bloating, and discomfort [1]. With a prevalence of 10%–20% worldwide, IBS accounts for up to 40% of all practice consultation referred to gastroenterologists [2]. Recurrent abdominal discomfort or pain is among the most typical complaints in IBS patients. Although the pathophysiology is elusive, it has been observed that visceral hypersensitivity is implicated in the development of IBS symptoms and is an important hallmark feature of IBS [1, 3, 4]. Visceral hypersensitivity which occurs in the nervous system has been found in 20%–90% of IBS patients while lower pain threshold to the colorectal stimuli was found in most IBS patients compared to the healthy controls [2, 4, 5].

Acetylcholine is a pivotal molecule in numerous physiological processes, and the cholinergic system has significant antinociceptive effects in the development of inflammatory, neuropathic, and visceral pain [6, 7]. It is reported that cholinergic system involves in the development of visceral hypersensitivity [8, 9]. Essential components in the cholinergic neurotransmission, including choline acetyltransferase, acetylcholine esterase, vesicular acetylcholine transporter, and nicotinic and muscarinic acetylcholine receptors, have been well studied. In the acetylcholine synthesis, choline is the precursor of acetylcholine, and choline uptake has been regarded as the rate-limiting step [10]. High-affinity choline transporter of choline uptake system, which is designated as CHT1, has been verified to be specifically expressed on the presynaptic terminals and involves in the choline uptake [10, 11]. Our previous study has showed that CHT1 is increased in the model of pancreatitis-induced pain, and CHT1 inhibitor can enhance the behavioral response to abdominal mechanical stimulation in rats [12]. However, the involvement of CHT1 in the noninflammatory visceral hypersensitivity induced by chronic stress remains unknown.

Dorsal root ganglion (DRG) is located at the junction of the peripheral and central nervous systems (CNSs) both anatomically and functionally [13]. It has been confirmed that the DRG involves in the modulation of chronic pain as well as visceral hypersensitivity [14–16]. Sensory information is transmitted from intestines to CNS via the afferent nerves of colon, whose cell bodies are located at the DRGs [17, 18]. Previous studies have found that pain regulatory pathways are dysregulated in DRG neurons in stress-induced visceral hyperalgesia models [19]. Thus, as a region which conveys and controls visceral sensation, DRG occupies a significant position and acts as a target of neuromodulation.

Moreover, chronic stress is related to visceral hypersensitivity and altered bowel function in human and animal models [20, 21], and water avoidance stress (WAS) is a validated method to investigate visceral hypersensitivity [22]. The responses to colorectal distension (CRD) reflect the degree of visceral hypersensitivity [18]. Thus, abdominal withdrawal reflex (AWR) and the threshold pressure which induced the first contraction of abdominal muscles in CRD test were recorded to estimate the level of visceral sensitivity.

Here, the rat model of visceral hypersensitivity was induced by repetitive WAS and was assessed by CRD test. The mRNA and protein expressions of CHT1 on DRG were also detected. After intraperitoneal injection of hemicholinium-3 (HC-3), the specific inhibitor of CHT1, CRD data were also recorded to evaluate the potential role of CHT1.

## 2. Material and Methods

### 2.1. Animals

All experiments were approved by the Animal Ethics Committee of Renmin Hospital, Wuhan University. Adult Sprague-Dawley rats (male, 180–220 g) were purchased from Hubei Province Center for Disease Control and Prevention. Animals were housed in a temperature-controlled environment (25 ± 1°C) of 12 h light/12 h dark cycle and were provided with food and water ad libitum.

Rats were randomly grouped and underwent WAS or sham stress exposure for 10 days as previous study [22]. Room temperature water (25°C) was filled in a Plexiglas tank. A platform was fixed in the tank and was 1 cm over the height of water. WAS rats were put on the platform for 1 h each day individually while the sham control rats were placed without water. Experiments were conducted on day 11 of the stress or sham exposure.

### 2.2. Measurement of Visceral Hypersensitivity

The behavioral response to the stimulation of CRD was determined to measure the degree of visceral sensitivity. AWR and threshold pressure of CRD which induced the first contraction of abdominal muscles were assessed in a blinded manner. As previous studies [18, 23], rats were anaesthetized by isoflurane using an anesthetic equipment (AS-01-0007, Summit, USA). Connected with syringe and sphygmomanometer, a balloon was inserted into the colorectum after lubrication. After 30 min recovery from anesthesia, we rapidly inflated the balloon to 20, 40, 60, and 80 mmHg. Each measurement was performed in triplicate, and the colorectum was distended with 30 s duration followed by a 5-minute interval. The AWR was measured with a semiquantitative scoring system by CRD as following: 0, no response; 1, immobility after brief head moving; 2, contractions of abdominal and hind limb musculature; 3, abdominal hunching; and 4, body arching and pelvis shaking [18]. The threshold pressure of CRD was identified as the pressure of the pain (AWR score ≥ 2) behavior displayed while the maximum was regarded as 80 mmHg to avoid unpredictable damage to animals. All the measurements were observed by two blinded observers.

0.01% HC-3 (Sigma, USA), the specific inhibitor of CHT1, was freshly dissolved in saline and injected intraperitoneally into the WAS and sham stress rats at the concentrations of 60 *μ*g/kg, 80 *μ*g/kg, or 100 *μ*g/kg. Normal saline was injected as control. Three hours after administration, the AWR score as well as the threshold intensity of CRD were recorded as the same method.

### 2.3. Immunohistochemistry (IHC)

Animals were anesthetized with 4% isoflurane and sacrificed. Bilateral colonic DRGs (T12 to S1) were dissected, fixed in 4% paraformaldehyde, and embedded in paraffin. All the specimens were cut into 5 *μ*m thick sections. After deparaffinization and rehydration, antigen retrieval was performed at microwave oven for 15 minutes in citrate buffer (0.01 mM, pH = 6.0). After blocking with 2% BSA and washing, sections were then incubated in the anti-CHT1 monoclonal antibody (mouse anti-rat IgG, 1 : 200, Santa Cruz Biotechnology, CA, USA) overnight at 4°C. PBS instead of the primary antibody served as negative control. After washing three times, sections were then incubated with HRP-conjugated antibody (goat anti-mouse IgG, 1 : 200, Boster, Wuhan, China) for 30 minutes and stained by 3,3′-diaminobenzidine (DAB) chromogenic reagent. Finally, counterstaining was performed with hematoxylin. The signals obtained from the labeling cells were detected via Olympus BX53 microscopy (CCD DP80).

### 2.4. Western Blotting

Equal protein (25 *μ*g) from bilateral colonic DRGs (T12 to S1) was separated by electrophoresis on 10% SDS-PAGE and then transferred to nitrocellulose membranes. After blocking with 2% BSA and washing, membranes were incubated with anti-CHT1 monoclonal antibody (mouse anti-rat IgG, 1 : 200, Santa Cruz, CA, USA) at 4°C overnight. Membranes were subsequently incubated for 2 hours with HRP-conjugated IgG antibody (goat anti-mouse IgG, 1 : 5000, Servicebio, Wuhan, China). Data was presented as band density which was normalized relative to GAPDH (1 : 1000, Servicebio, Wuhan, China).

### 2.5. Quantitative RT-PCR

Total RNA was extracted from colonic DRGs (T12 to S1) with an RNA extraction kit (Invitrogen, USA). 2.0 *μ*g of total RNA from WAS group and sham stress group was loaded for cDNA synthesis. ABI 7500 real-time PCR system (Thermo Fisher, CA, USA) was used in the detection. Quantitative RT-PCR was carried out in 20 *μ*l wells with SYBR Premix Ex Taq II (Takara, Otsu, Japan). The reaction condition was 95°C incubation to denature for 10 minutes, amplification for 40 cycles (95°C for 5 seconds followed by 60°C for 30 seconds). All samples were tested in triplicate. The primers of CHT1 were forward 5′-GACTGTGTATGGGCTCTGGT-3′ and reverse 5′-TGGCTCTCCTCCGGTAATTC-3′. Each sample was normalized with GAPDH. The 2^−∆∆Ct^ method was used to calculate relative transcript level of the CHT1.

### 2.6. Detection of Acetylcholine Concentration

Fresh DRGs were removed from rats. After homogenization on ice and centrifugation (12,000*g* for 10 min at 4°C), the supernatant was collected to measure the acetylcholine concentration by the acetylcholine/acetylcholinesterase assay kit (Invitrogen). Each sample was assayed in triplicate.

### 2.7. Statistical Analysis

All data are presented as mean ± standard deviation. Statistical analyses were carried out in Prism 5.00 software (GraphPad, USA), and the significant difference was determined by two-sample *t*-test or one-way analysis of variance (ANOVA) and post hoc Tukey's test as appropriate. The comparison was considered to be statistically significant when *P* values < 0.05.

## 3. Results

### 3.1. WAS Induces Visceral Hypersensitivity

**Figure 1:**
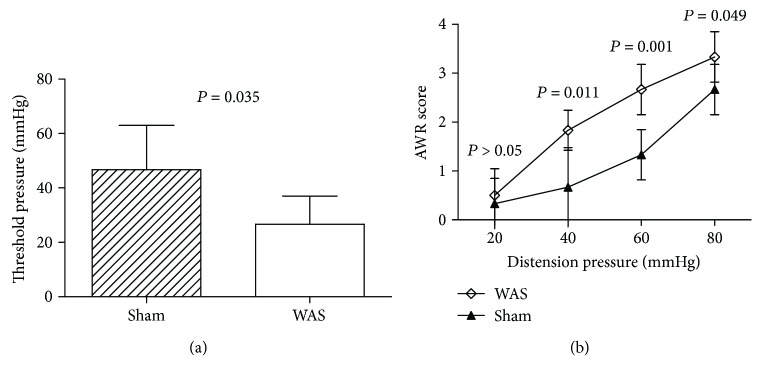
Measurement of visceral hypersensitivity between water avoidance stress (WAS) group and sham stress group. (a) The threshold pressures in response to colorectal distension (CRD). *P* = 0.035 analyzed by two-sample *t*-test, *n* = 6 per group. (b) Abdominal withdrawal reflex (AWR) scores in response to CRD. *P* > 0.05, *P* = 0.045, 0.046, and 0.047 at different distention pressure by two-sample *t*-test, compared to the sham stress group, *n* = 6 per group.

AWR score and threshold pressure to CRD reflected the responses of visceral hypersensitivity. After 10 days of WAS, the mean threshold of CRD was significantly lower (*P* = 0.035, Figure 1(a)), and the AWR score was significantly higher at the distension pressures of 40, 60, and 80 mmHg compared to the sham stress rats (*P* = 0.011, 0.001, and 0.049, resp., Figure 1(b)). The mean AWR scores at 20 mmHg were higher in WAS group than the sham stress group, but not statistically (*P* > 0.05, Figure 1(b)).

### 3.2. WAS Increased CHT1 Expression in DRG

**Figure 2:**
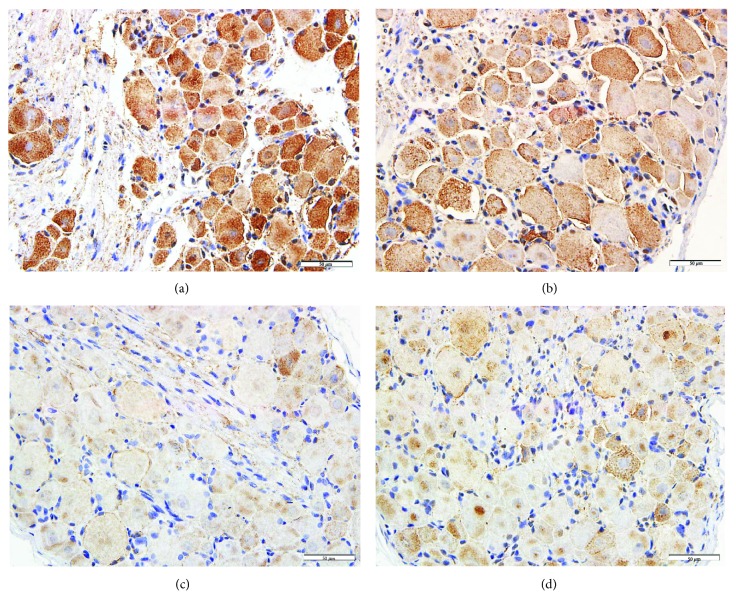
Immunohistochemistry analysis of CHT1 expression in dorsal root ganglion (DRG). Immunohistochemistry revealed an enhancement of CHT1 staining in the water avoidance stress group (a, b) compared to the sham stress group (c, d). Scale bar = 50 mm.

**Figure 3:**
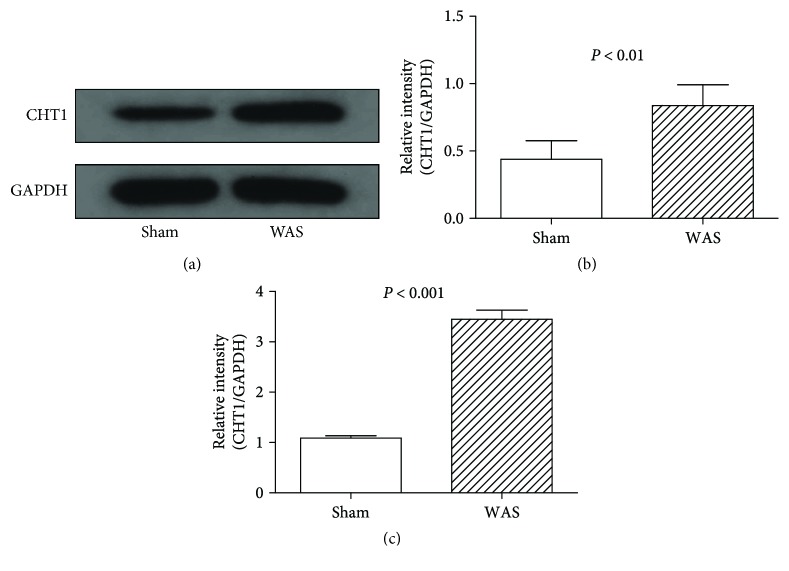
Western blotting and quantitative RT-PCR analysis of CHT1 expression in dorsal root ganglion (DRG). (a) Representative Western blotting for CHT1 in colonic DRGs. (b) Levels of CHT1 protein measured by Western blotting analysis. *P* < 0.01 by two-sample *t*-test, *n* = 6 per group. (c) Levels of CHT1 expression measured by quantitative RT-PCR. *P* < 0.001 by two-sample *t*-test, *n* = 6 per group.

As showed in Figure 2, CHT1 detected by IHC was located at the neurons of DRG. WAS group showed enhanced CHT1 staining compared to the sham stress group (Figures 2(a)–2(d)). The results of Western blotting demonstrated that after 10 days of WAS, the CHT1 expression of DRG was dramatically increased compared to the sham stress rats (*P* < 0.001, Figures 3(a) and 3(b)).

Further quantitative RT-PCR analysis confirmed that CHT1 expression in DRG of WAS group was dramatically higher compared to the sham stress group at the transcriptional level (*P* < 0.001, Figure 2(c)). These results illustrated that repetitive exposure to WAS may lead to the CHT1 overexpression at mRNA and protein levels in the DRG.

### 3.3. Inhibition of CHT1 Enhances the Visceral Hypersensitivity

**Figure 4:**
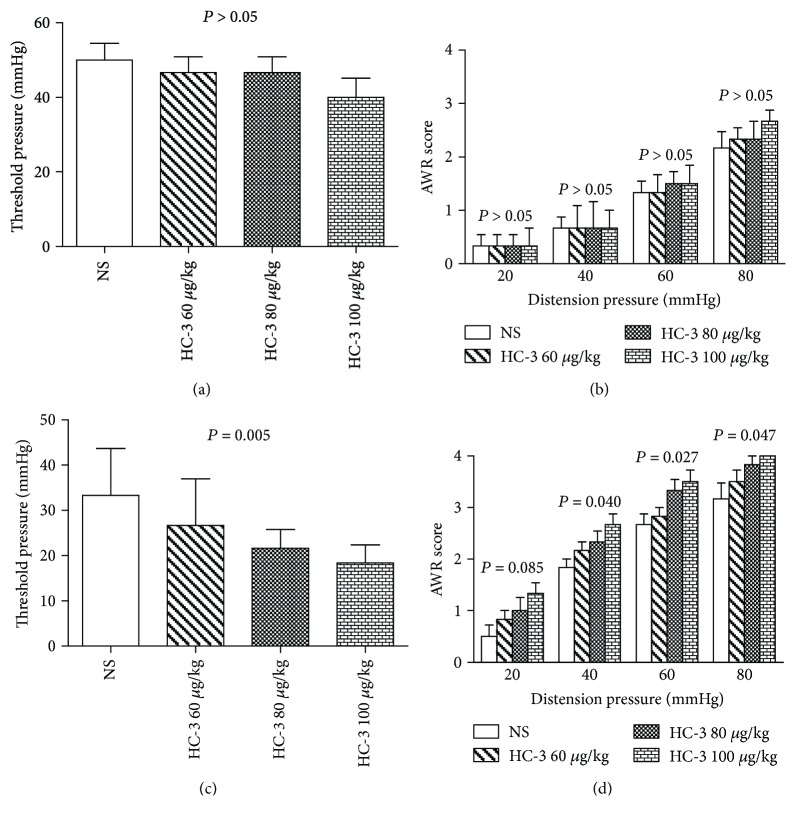
Measurement of visceral hypersensitivity in the sham stress group and water avoidance stress (WAS) group after the administration of hemicholinium-3 (HC-3). (a) The threshold pressures in the sham stress group. *P* > 0.05 by one-way analysis of variance, *n* = 6 per group. (b) Abdominal withdrawal reflex (AWR) scores in the sham stress group. *P* > 0.05 at each distention pressure by one-way analysis of variance, *n* = 6 per group. (c) The threshold pressures in the WAS group. *P* = 0.005 by one-way analysis of variance, *n* = 6 per group. (d) AWR scores in the WAS group. *P* = 0.085, 0.040, 0.027, and 0.047 at different distention pressure by one-way analysis of variance, *n* = 6 per group.

In order to investigate the contribution of CHT1 to visceral hypersensitivity, HC-3, the specific inhibitor of CHT1, was injected intraperitoneally into the WAS rats and the sham stress rats. In the sham stress group, the threshold intensity and the AWR score of CRD showed no significant differences among the normal saline group and HC-3 groups (*P* > 0.05, Figures 4(a) and 4(b)). While in WAS group, it was found that the threshold intensity of CRD showed remarkably difference among different doses of HC-3 and normal saline groups (*P* = 0.005, Figure 4(c)). Meanwhile, the AWR score was significantly increased compared to the normal saline control at the distention pressures of 40, 60, and 80 mmHg (*P* = 0.040, 0.027, and 0.047, resp., Figure 4(d)).

### 3.4. Inhibition of CHT1 Depletes the Acetylcholine Concentration

**Figure 5:**
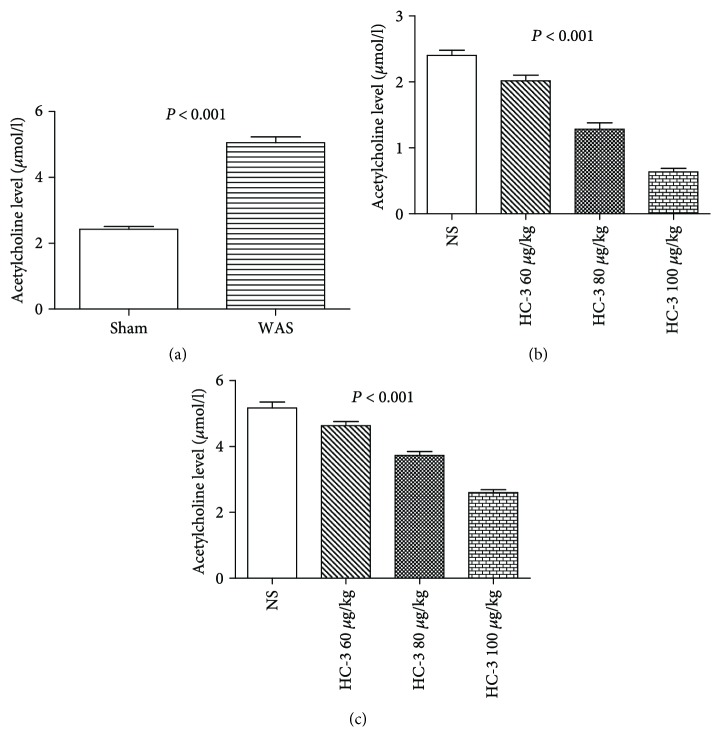
Acetylcholine concentration in DRG tissues of sham stress group and water avoidance stress (WAS) group. Acetylcholine content was significantly reduced in a dose-dependent manner after the injection of HC-3 in both sham and WAS groups. (a) Acetylcholine content in sham stress group and WAS group. *P* < 0.01 by two-sample *t*-test, *n* = 6 per group. (b) Acetylcholine content in sham stress group after injection of HC-3. *P* = 0.005 by one-way analysis of variance, *n* = 6 per group. (c) Acetylcholine content in WAS group after injection of HC-3. *P* = 0.005 by one-way analysis of variance, *n* = 6 per group.

Acetylcholine concentration was detected before and after HC-3 injection. Before the HC-3 was injected, acetylcholine level was dramatically higher in WAS group than the sham control (*P* < 0.001, Figure 5(a)), and the content of acetylcholine was significantly reduced in a dose-dependent manner after the injection of HC-3 in both sham stress (*P* < 0.001, Figure 5(b)) and WAS groups (*P* < 0.001, Figure 5(c)).

## 4. Discussion

The exact mechanisms of IBS are poorly understood, and effective therapeutics for the primary symptoms remains difficult [24]. As excessive response to colorectal stimuli, visceral hypersensitivity has been regarded as one of the most important causes of abdominal pain in IBS [25]. In this study, a visceral hypersensitivity model of rats was established by repetitive WAS method, and the overexpression of CHT1 in the colonic DRG was confirmed. Additionally, we found that CHT1 participated in the pathophysiological processes of visceral hypersensitivity.

WAS model simulates the repetitive daily exposure of chronic stress underwent by humans and has been considered as an effective model to investigate the potential mechanisms of visceral hypersensitivity [22]. Here, we successfully induced visceral hypersensitivity model of rat by chronic stress for 10 days. As quantitative criteria of visceral hypersensitivity, CRD can induce the contraction of abdominal muscles in rats [26, 27]. Based on the enhancement of the abdominal muscle contraction, AWR score and threshold intensity of CRD were used to measure the visceral hypersensitivity. In our study, the WAS group showed significantly higher AWR score at high distension pressure and lower threshold intensity of CRD than the sham stress group. Our results demonstrated that WAS rats suffered from visceral hypersensitivity, which is consistent with the previous study.

The CHT1 expression level of visceral hypersensitivity model was further investigated. Acetylcholine has significant antinociceptive effects in the development of visceral pain. Meanwhile, the enzyme inhibitors and receptor agonists of acetylcholine in antinociception have been successfully used in the pain signaling inhibition [7]. The high-affinity choline transporter CHT1 also known as solute carrier family 5 member 7 belongs to the Na^+^/glucose cotransporter family, which is a cell membrane transporter and mediates the choline uptake of acetylcholine synthesis in cholinergic nerve terminals [28–30]. In the central and peripheral nervous systems, CHT1 is expressed almost exclusively in cholinergic neurons [31–34]. Previous studies show that CHT1 dysfunction is mainly related to neurological and psychiatric disorders, such as Alzheimer's disease [35, 36], Huntington's disease [37, 38], and attention deficit hyperactivity disorder [39]. Nevertheless, few studies have addressed how CHT1 is involved in the development of visceral hypersensitivity.

In the present study, we firstly elucidated that CHT1 was overexpressed in the colonic DRG of WAS rats compared to sham stress group, which suggested that CHT1 expression is associated with visceral hypersensitivity. HC-3, a specific inhibitor of CHT1, was injected in rats to explore the exact role of CHT1. After administration in WAS rats, the HC-3 groups showed lower threshold intensity and higher AWR score of CRD than the normal saline group in a dose-dependent manner. It is worth noting that in the sham stress group, the threshold intensity and AWR score showed no significant differences after HC-3 injection compared to the normal saline group, which indicates that the blockage of CHT1 may not lead to nociception in normal rats.

Acetylcholine level was dramatically higher in WAS group than the sham control, which is consistent with the previous study [12] and indicates that acetylcholine may be released in the inflammatory or noninflammatory algesia models and mediate the antinociceptive effect. The content of acetylcholine was significantly reduced in a dose-dependent manner after the injection of HC-3 in both sham and WAS groups. Taken together, these results demonstrate that CHT1 plays an antinociceptive role in visceral hypersensitivity by increasing the acetylcholine level while inhibition of CHT1 can enhance the visceral hypersensitivity.

Moreover, CHT1 has its advantages on pain treatment compared to other cholinomimetics. On the one hand, acetylcholinesterase, muscarinic acetylcholine receptors, and nicotinic acetylcholine receptors, which are efficacious in different preclinical and clinical pain models, can cause profound cardiovascular, respiratory, gastrointestinal, and even central side effects and death [7]. On the other hand, uptake of high-affinity choline is rate-limiting for the synthesis of acetylcholine, which indicates that CHT1 has a prospective future in pain control [40].

In conclusion, our research demonstrates that visceral hypersensitivity induced by repetitive WAS in rats is associated with the increased CHT1 expression in DRG, and blockage of CHT1 can enhance the visceral hypersensitivity, suggesting CHT1 may be a potential therapeutic target of IBS and related disorders.

## Data Availability

The data used to support the findings of this study are available from the corresponding author upon request.
